# Application of Liquid Chromatography in Food Analysis

**DOI:** 10.3390/foods9091277

**Published:** 2020-09-11

**Authors:** Oscar Núñez, Paolo Lucci

**Affiliations:** 1 Department of Chemical Engineering and Analytical Chemistry, University of Barcelona, Martí i Franquès 1-11, E08028 Barcelona, Spain; 2 Research Institute in Food Nutrition and Food Safety, University of Barcelona, Recinte Torribera, Av. Prat de la Riba 171, Edifici de Recerca (Gaudí), Santa Coloma de Gramenet, E08921 Barcelona, Spain; 3 Serra Húnter Fellow, Generalitat de Catalunya, Rambla de Catalunya 19-21, E08007 Barcelona, Spain; 4 Department of Agri-Food, Environmental and Animal Sciences, University of Udine, 33100 Udine, Italy

Food products are very complex mixtures consisting of naturally-occurring compounds and other substances, generally originating from technological processes, agrochemical treatments, or packaging materials. Several of these compounds (e.g., veterinary drugs, pesticides, mycotoxins, etc.) are of particular concern because, although they are generally present in very small amounts, they are nonetheless often dangerous to human health. On the other hand, food is no longer just a biological necessity for survival. Society, in general, demands healthy and safe food, but it is also increasingly interested in other quality attributes more related to the origin of the food, the agricultural production processes used, the presence or not of functional compounds, etc. In an increasingly populated world and with an increasingly demanding society regarding food quality, food production has become a completely global aspect on a global level. In addition, in this field, where the number of people involved in the food production process, from its origin to its consumption, is enormous, it is increasingly difficult to guarantee the integrity and, above all, the authenticity of foodstuffs. In consequence, improved methods for the determination of authenticity, standardization, and efficacy of nutritional properties in natural food products are required to guarantee their quality and for the growth and regulation of the market. Thus, food safety and food authentication are hot topics for both society and the food industry.

Nowadays, liquid chromatography with ultraviolet (LC-UV) detection, or coupled to mass spectrometry (LC-MS) and high-resolution mass spectrometry (LC-HRMS), are among the most powerful techniques to address food safety issues and to guarantee food authenticity in order to prevent fraud [1,2,3,4,5,6,7,8]. The aim of this Special Issue “Application of Liquid Chromatography in Food Analysis” was to gather review articles and original research papers focused on the development of analytical techniques based on liquid chromatography for the analysis of food. This Special Issue is comprised of six valuable scientific contributions, including five original research manuscripts and one review article, dealing with the employment of liquid chromatography techniques for the characterization and analysis of feed and food, including fruits, extra virgin olive oils, confectionery oils, sparkling wines and soybeans.

Cortés-Herrera et al. reviewed the potential of liquid chromatography for the analysis of common nutritional components in feed and food [9]. Food and feed share several similarities when facing the implementation of liquid-chromatographic analysis. Using the experience acquired over the years through the application chemistry in food and feed research, the authors selected and discussed analytes of relevance for both areas. This interesting review addresses the common obstacles and peculiarities that each analyte offers for the implementation of LC methods throughout the different steps of the method development (sample preparation, chromatographic separation and detection). The manuscript consists mainly of three sections: feed analysis (at the beginning of the food chain); food destined for human consumption determinations (the end of the food chain); and finally, assays shared by either matrices or laboratories. Polyphenols, capsaicinoids, theobromine and caffeine, cholesterol, mycotoxins, antibiotics, amino acids, triphenylmethane dyes, nitrates/nitrites, ethanol soluble carbohydrates/sugars, organic acids, carotenoids, and hydro and liposoluble vitamins are examined. 

Several original research works reported the application of liquid chromatography-based analytical methodologies for the characterization of food products. Loizzo et al. characterized native Colombian fruits and their by-products by determining their phenolic profile, antioxidant activity and hypoglycaemic potential [10]. The use of ultra-high performance liquid chromatography-high resolution mass spectrometry (UHPLC-HRMS) with an Orbitrap mass analyzer revealed the presence of chlorogenic acid as dominant compound in Solanaceae samples. In addition, and based on the Relative Antioxidant Score (RACI) and Global Antioxidant Score (GAS) values, *Solanum quitoense* peel showed the highest antioxidant potential among Solanaceae samples, while *Passiflora tripartita* fruits exhibited the highest antioxidant effects among Passifloraceae samples. Considering that some of the most promising results were obtained by the processing waste portion, the authors highlighted that its use as functional ingredients should be considered for the development of nutraceutical products intended for patients with disturbance of glucose metabolism. Obyedul Kalam Azad et al. evaluated the effect of artificial LED light and far infrared (FIR) irradiation on phenolic compounds, isoflavones and the antioxidant capacity of soybean (*Glycine max* L.) sprouts [11]. Six isoflavones (daidzin, glycitin, genistin, daidzein, glycitein and genistein) were determined by LC. The authors applied artificial blue (470 nm) and green (530 nm) LED and florescent light (control) on soybean sprouts, from three to seven days after sowing in the growth chamber. Total phenolic content, antioxidant capacity and total isoflavones content were higher under blue LED compared to control. Thus, results suggested that blue LED was the most suitable light to steady accumulation of secondary metabolites in growing soybean sprouts. In another interesting research work, polyphenolic profiles obtained by high-performance liquid chromatography with ultraviolet/visible detection (HPLC-UV/Vis) and principal component analysis (PCA) were employed by Izquierdo-Llopart and Saurina for the characterization of sparkling wines [12]. Chromatographic profiles were recorded at 280, 310 and 370 nm to gain information on the composition of benzoic acids, hydroxycinnamic acids and flavonoids, respectively. The authors employed the obtained HPLC-UV/vis data, consisting of composition profiles of relevant analytes, to characterize cava wines produced from different base wine blends by using chemometrics. Other oenological variables, such as vintage, aging or malolactic fermentation, were fixed over all the samples to avoid their influence on the description. PCA and other statistic methods were able to extract the underlying information and provided an excellent discrimination of the analyzed samples according to their grape varieties and coupages. Finally, Santoro et al. performed the characterization and determination of interesterification markers (triacylglycerol regioisomers) in confectionery oils by liquid chromatography-high resolution mass spectrometry (LC-HRMS) [13]. In the confectionery industry, controlling the formation degree of positional isomers is important in order to obtain fats with the desired properties. The separation of triacylglycerol regioisomers is a challenge when the number of double bonds is the same and the only difference is in their position within the triglyceride molecule. The authors aimed to obtain a chromatographic resolution that might allow reliable qualitative and quantitative evaluation of triacylglycerol positional isomers within rapid retention times, and robustness in respect of repeatability and reproducibility by means of LC-HRMS using an LTQ-Orbitrap analyzer with atmospheric pressure chemical ionizacion (APCI). The time required for the global analysis was relatively short, the chromatographic resolution and efficiency were satisfactory and the mass detection allowed for identifying the isobaric components of each position isomer couple. In conclusion, the described method may well be considered a good diagnostic tool of interesterification consequences that are strictly connected to confectionery product quality.

HPLC-UV was also proposed by Carranco et al. for the authentication and quantitation of frauds in extra virgin olive oils (EVOOs) [14]. For that purpose, HPLC-UV chromatographic fingerprints recorded at 257, 280 and 316 nm were employed as sample chemical descriptors for the characterization and authentication of monovarietal EVOOs and other vegetable oils by chemometrics. PCA results showed a noticeable discrimination between olive oils and other vegetable oils using raw HPLC-UV fingerprints as data descriptors. However, the authors observed that selected HPLC-UV chromatographic time-window segments were able to improve the discrimination among the monovarietal EVOOs analyzed. In addition, partial least squares (PLS) regression was employed to tackle olive oil authentication of Arbequina EVOO adulterated with Picual EVOO, refined olive oil, and sunflower oil, achieving highly satisfactory results with overall errors in the quantitation of adulteration in the Arbequina EVOO (minimum 2.5% of adulterant) below 2.9%. 

In summary, the Special Issue “Application of Liquid Chromatography in Food Analysis” demonstrated the great importance of liquid chromatography analytical methodologies to address the characterization and determination of targeted compounds in food, as well as to guarantee food integrity and authenticity. In addition, several authors have also demonstrated the requirement of advanced chemometric approaches as essential tools in combination with LC to achieve robust results, not only with the objective of characterizing food composition, but also to obtain satisfactory classification methods, and to prevent food fraud.
